# Analgecine enhances the anti-tumor response of radiotherapy by increasing apoptosis and cell cycle arrest in non-small cell lung cancer

**DOI:** 10.18632/oncotarget.19968

**Published:** 2017-08-07

**Authors:** Xue Chen, Xibing Zhuang, Qi Zhang, Youjun Luo, Sujuan Yuan, Tiankui Qiao

**Affiliations:** ^1^ Department of Oncology, Jinshan Hospital, Medical Center of Fudan University, Jinshan District, Shanghai 201500, People's Republic of China

**Keywords:** analgecine, apoptosis, cell cycle, non-small cell lung cancer, radiotherapy

## Abstract

We investigated whether Analgecine treatment enhanced the antitumor response of radiotherapy in non-small cell lung cancer (NSCLC) cells. Lewis lung carcinoma (LLC) xenograft mice treated with Analgecine plus irradiation showed reduced tumor growth and increased survival. Tumor cell apoptosis was enhanced by Analgecine, based on TUNEL assays. It also increased plasma levels of pro-inflammatory cytokines (IL-6, IL-12, and IFN-γ) and decreased anti-inflammatory cytokines (TGFβ and IL-10), suggesting an enhanced immune response. Analgecine plus irradiation reduced cell viability and colony formation by A549 NSCLC cells. Analgecine treatments also activated apoptotic signaling with increased levels of pro-apoptotic proteins, including cytochrome c, caspase-3, cleaved caspase-3, caspase-9, p53 and Bax, and decreased Bcl2. Analgecine enhanced G2/M phase arrest in A549 cells by decreasing cyclinB1 and CDK1. These observations demonstrate that Analgecine combined with radiotherapy enhances anti-tumor responses by inducing apoptosis and cell cycle arrest. Moreover, they suggest possible future clinical application of Analgecine for the treatment of NSCLC.

## INTRODUCTION

Lung cancer is a leading cause of cancer deaths worldwide and non-small cell lung cancer (NSCLC) accounts for nearly 85% of all lung cancers [1]. Radiotherapy is one of the main approaches for locally advanced NSCLC patients that are inoperable or unwilling to undergo surgery [2]. Radiotherapy plays a vital role in regulating inflammatory factors and immune functions [3, 4]. The ionizing radiations (IR) enhance antitumor response by inducing apoptosis in tumor cells [5]. However, radiotherapy with high-dose ionizing radiation is also associated with adverse effects including neural damage, chronic pain and radioresistance [3, 6]. Also, IR affects inflammatory factors in the tumor microenvironment, resulting in chronic pain that impacts the quality of life for most patients [7, 8]. Therefore, there is urgent need for strategies to overcome the adverse effects due to radiotherapy. In some studies, adjuvant therapies have been used to improve the IR response and reduce IR injury to reduce chronic pain [9–11]. However, adverse side effects of the adjuvant therapy have restricted their use in the clinic. Therefore, newer therapeutics that enhances adjuvant treatment safety and efficacy are urgently needed.

Analgecine is a non-protein extract from inflamed rabbit skin inoculated with vaccinia virus that has been widely used for clinical applications in China to treat chronic pain conditions [12]. Its pharmacological effects include nerve repair, analgesia and immune function regulation. For example, Analgecine is usually used to treat cancer pain, post herpetic neuralgia, lower back pain, cervicodynia and peripheral neuropathies [13–15]. Moreover, clinical trials and animal experiments have demonstrated that Analgecine is effective in controlling neural damage and chronic pain [14, 16]. Liu *et al.* demonstrated that Analgecine reduced oxaliplatin-induced neurotoxicity in gastrointestinal cancer patients [14]. Furthermore, a randomized multicenter double-blind placebo-controlled phase 3 clinical trial showed that Analgecine effectively alleviated chronic lower back pain with fewer adverse effects [12]. Although the therapeutic effect of Analgecine on chronic pain has been established for decades, the effect of Analgecine in combination with radiotherapy against tumors has not been investigated. Therefore, we investigated if Analgecine in combination with radiotherapy may enhance the antitumor response by using the Lewis lung carcinoma (LLC) *in vivo* mouse model and in A549 cells *in vitro*.

## RESULTS

### Analgecine reduces xenograft tumor growth and enhances mice survival

**Figure 1:**
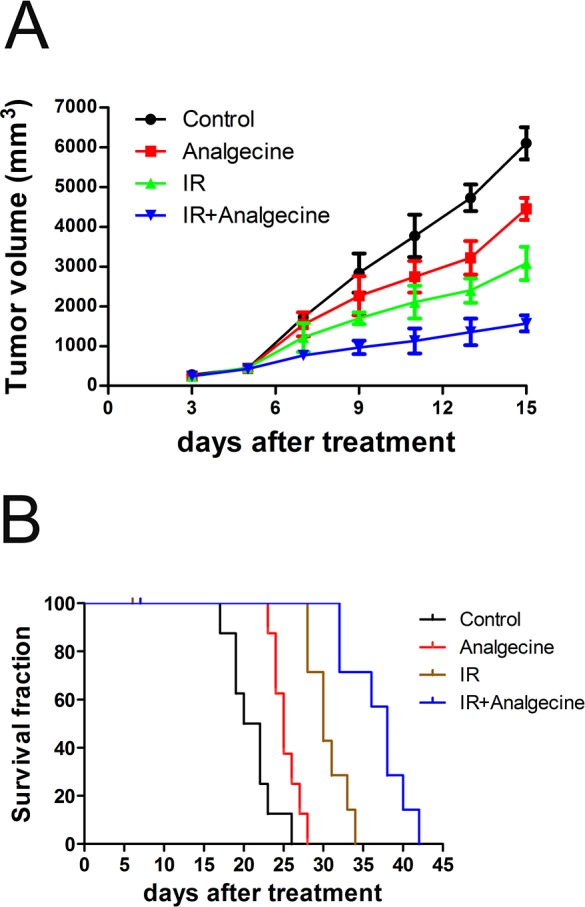
Analgecine in combination with IR inhibits tumor growth and enhances survival C57BL/6 mice bearing xenografted Lewis lung carcinoma (LLC) cells were divided into four groups: the control (NS), Analgecine(0.012U/g), IR (3Gy), and IR+Analgecine (combination). **(A)** Tumor growth was significantly reduced at day 7 in combination treatment group mice compared to the controls (P<0.05). At day 9, all 3 treatment groups demonstrated reduced tumor growth compared to the control (P<0.05). **(B)** Kaplan Meier survival curves of the 4 treatment groups of mice. The combination treatment showed increased survival of xenografted mice compared to the other 3 groups (P<0.001). All experiments were performed in duplicate.

We investigated the therapeutic effects of Analgecine alone or in combination with radiotherapy using the xenograft LLC mice model. As shown in Figure 1A, tumor growth was comparable in all 4 groups on day 5. Further, we observed decreased tumor growth in the combination treatment group compared to the other groups on days 7 (P<0.01) and day 9 (P<0.05). At 18 days, tumor growth in all 4 groups was comparable (data not shown).

The Kaplan Meier survival analysis demonstrated that treatment with Analgecine alone or in combination with IR prolonged the survival of mice (Figure 1B). The median survival times were 21, 25, 30, and 38 days for the control, Analgecine, IR, and Analgecine plus IR combination groups, respectively. This suggested synergistic anti-tumor effects between Analgecine and IR. During the survival analysis, one mouse each from IR group on day 6 and from combination group on day 7 died because of excessive anesthesia during the IR. Inspite of this, the antitumor response was most effective in the combination group with one mouse surviving until day 42.

### Analgecine increases xenograft tumor cell apoptosis and growth inhibition

**Figure 2:**
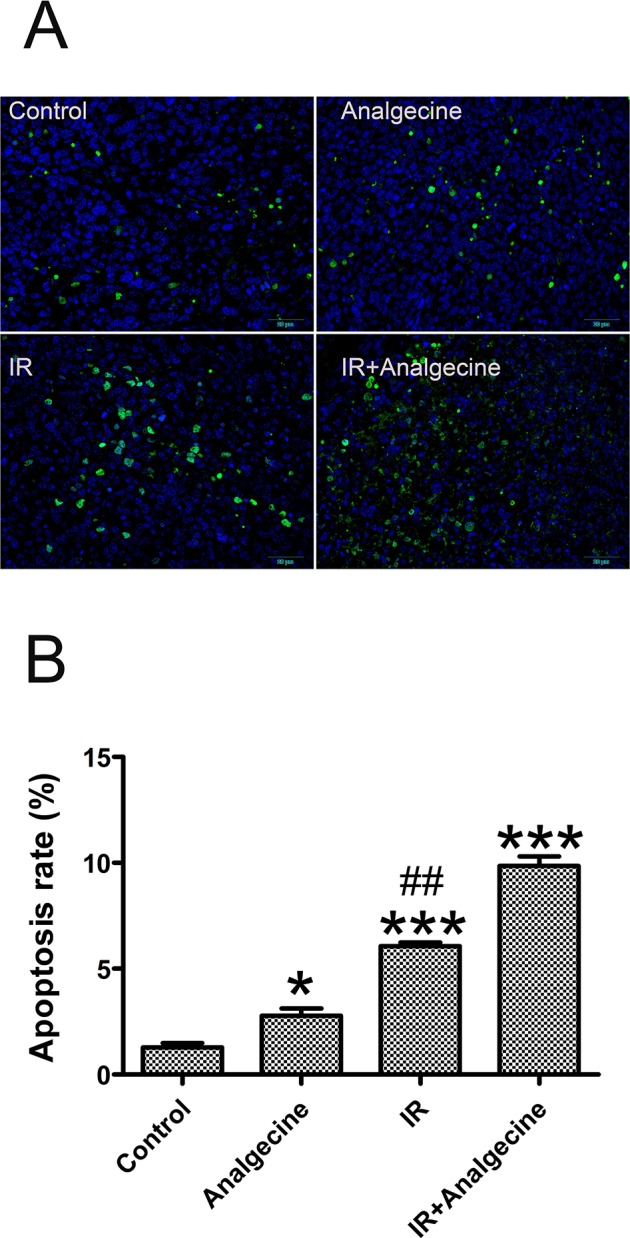
Analgecine in combination with IR enhances apoptosis in the xenograft LLC tumors **(A)** Representative immunofluorescence image of the TUNEL assay, showing apoptotic cells in green and the cell nuclei stained with DAPI in blue. Scale bar, 50 μm. **(B)** The tumor cell apoptosis rate in the tumors from all 4 groups was analyzed and the average numbers of TUNEL-positive cells (green) are shown for each group. *P<0.05 and ***P<0.001 denote statistical differences compared with the control group; ##P<0.01 denotes statistical difference compared with the combination group.

Next, we explored the effects of Analgecine on tumor cell apoptosis by TUNEL assay in the tumor tissues isolated from the 4 groups of mice. We observed increased apoptosis in Analgecine (P<0.05), IR (P<0.001) and Analgecine plus IR (P<0.001) groups compared to the control group (Figure 2A, 2B). Moreover, we observed increased apoptosis in the Analgecine plus IR group than IR group alone (P<0.01, Figure 2B), thereby demonstrating that the combination treatment was more effective. The tumors from the 4 groups of mice were weighed and the tumor inhibition rate was determined [tumor inhibition rate = 1-(mean tumor weight of treated group/mean tumor weight of control group) ×100%]. As shown in Table 1, the tumor inhibition rate gradually increased in the Analgecine, IR, and combination groups compared with the control group. Moreover, the tumor inhibition rate in the combination treatment group was higher than the IR group (P<0.05). This further demonstrated that the antitumor response of the combination therapy was more effective than IR alone.

**Table 1:** Tumor inhibition rate in analgecine and IR treatment groups. (mean ± standard deviation)

Group	Mice number	Tumor weight (g)	Tumor inhibition rate (%)
Control	4	7.56±0.90	
Analgecine	4	6.17±0.59*	18.3
IR	4	5.39±1.01*	28.63
IR+Analgecine	4	2.72±1.09***#	63.98

Tumor tissue was obtained from each group at day 13. Tumor inhibition rate=1-(mean tumor weight of treated group/mean tumor weight of control group)×100%. The data are shown as mean±standard deviation in each group. *P<0.05 and ***P<0.001 compared with control; #P<0.05 for the combination group compared with the IR group. The experiments were performed in duplicate.

### Analgecine enhances immune function in LLC tumor xenograft mice

**Figure 3:**
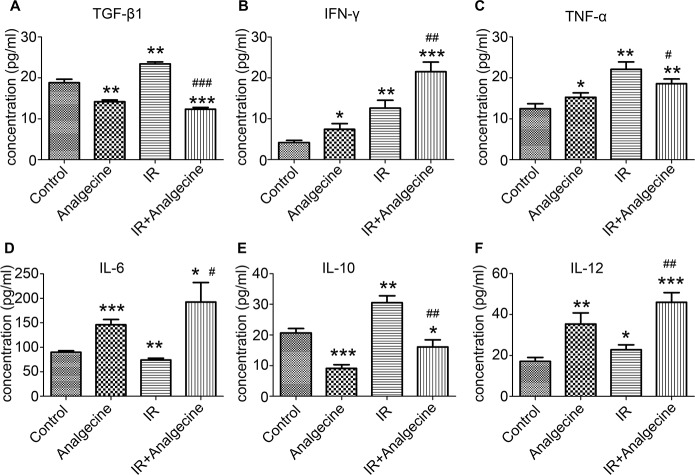
Analgescine treatment with IR enhances proinflammatory cytokines and reduces inhibitory cytokines in the LLC tumor xenograft mice **(A)** The plasma concentration of TGF-β1. **(B)** The plasma concentration of IFN-γ. **(C)** The plasma concentration of TNF-α. (**D**) Concentration of IL-6 in the plasma. **(E)** Concentration of IL-10 in the plasma. **(F)** Concentration of IL-12 in the plasma. The plasma concentration of various cytokines was determined using a mouse cytokine magnetic bead panel. Plasma levels of the immune inhibitory cytokines TGF-β1 and IL-10 were increased in IR treatment group, but decreased in combination treatment group. The levels of pro-inflammatory cytokines such as IL-6, TNF-α, and IL-12 were increased in combination treatment group compared to the control treatment group. *P<0.05, **P<0.01, ***P<0.001 denote statistical differences compared with the control group. #P<0.05, ##P<0.01 denote statistical differences compared with the IR group.

Next, we determined the effects of Analgecine on immune function by analyzing multiple cytokines and chemokines in the 4 groups of mice. We observed decreased TGF-β levels in the Analgecine and Analgecine plus IR treatment groups, whereas TGF-β levels were increased in the IR group compared to the control (P<0.01; Figure 3A). On the other hand, Analgecine, IR and Analgecine plus IR treatments demonstrated elevated IFN-γ levels (Figure 3B). TNF-α expression was upregulated in IR compared to the control (P<0.01, Figure 3C), but downregulated in Analgecine plus IR treatment group (P<0.05). IL-6 expression was decreased in the IR group, but increased in the Analgecine plus IR group (Figure 3D). The inhibitory cytokine IL-10 levels were lower in the Analgecine plus IR group compared to the control group (P<0.05, Figure 3E). However, IR treatment increased IL-10 levels compared to the control and combination treatments (P<0.01; Figure 3E). IL-12 levels were increased in the Analgecine, IR and Analgecine plus IR treatments (P<0.01 for Analgecine alone; P<0.001 for combination; Figure 3F).

### Analgecine decreases viability of irradiated A549 cells

**Figure 4:**
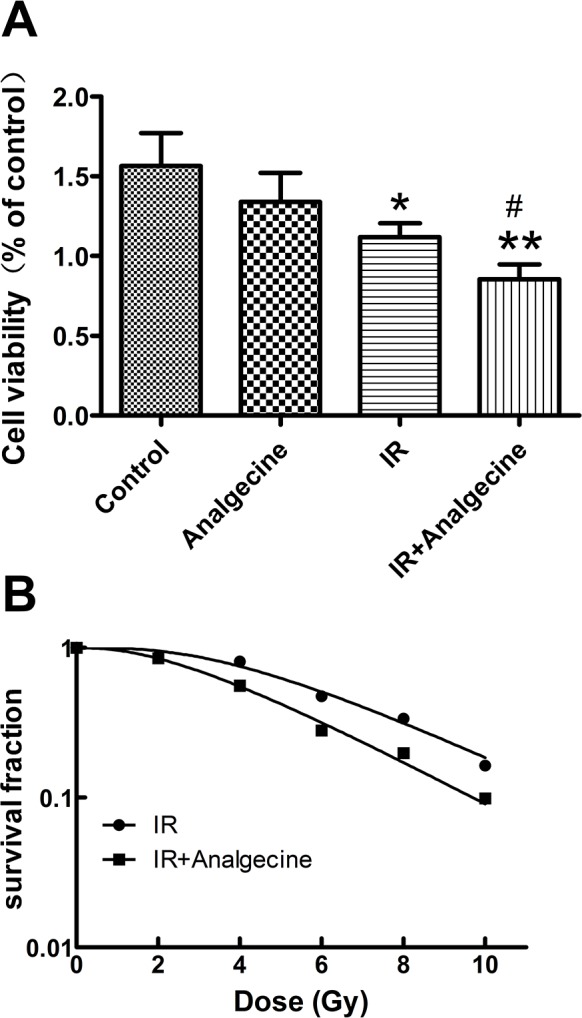
A549 cells treated with Analgescine and IR have reduced viability and colony formation ability **(A)** Cell viability of A549 cells was analyzed by CCK-8 assay at 24h. Analgecine in combination with IR significantly decreased cell viability compared to control or IR groups. *P<0.05, **P<0.01 denote significant differences compared with the control. #P<0.05 denotes significant difference compared with irradiation group. **(B)** Colony formation was performed to analyze the effect of Analgecine on radiosensitivity. Analgecine plus IR treatment significantly reduced the number of colonies compared with IR and control groups at all doses. The sensitivity enhancement ratio (SER) for combination group was 1.35. The D0 and N for the combination therapy was 2.998 and 2.614, while the D0 and N for IR alone was 4.058 and 2.483. The data are representative of mean±SD of triplicate experiments.

Next, we tested the effect of Analgecine on the viability of A549 cells. The combination treatment demonstrated significant decrease in cell viability compared to control (P<0.01) and IR (P<0.05) treatments based on CCK-8 assay (Figure 4A).

### Analgecine decreases colony formation ability of irradiated A549 cells

The colony-formation assay was performed to determine the effect of Analgecine on radiosensitivity of A549 cells. We observed that total numbers of colonies in the Analgecine plus IR group were reduced compared to other treatment groups (Figure 4B). The sensitivity enhancement ratio (SER) was 1.35.

### Analgecine enhances apoptosis and induces G2/M cell cycle arrest in irradiated A549 cells

**Figure 5:**
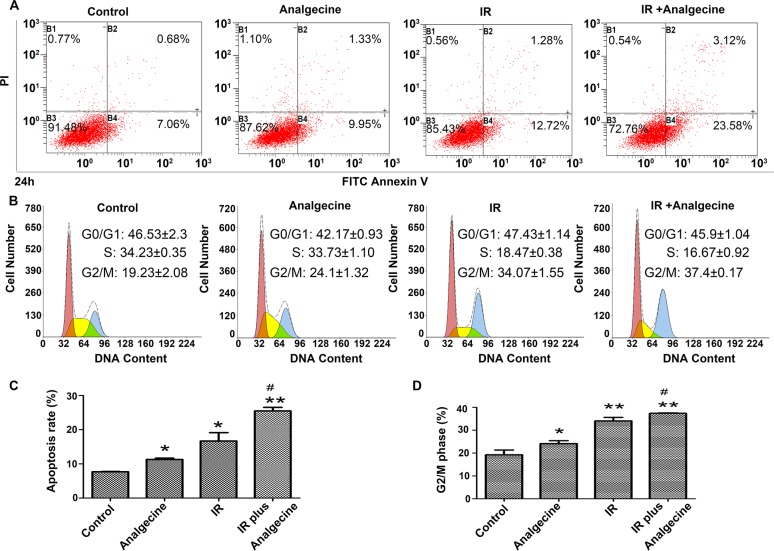
A549 cells treated with Analgecine and IR show increased apoptosis and G2/M cell cycle arrest **(A)** Representative FACS plots of AnnexinV/PI stained A549 cells that were treated with Analgecine alone or in combination with IR for 24h. **(B)** Representative FACS plots of cell cycle distribution of A549 cells stained with PI after treatment with Analgecine alone or in combination with IR for 24h. **(C)** Total percentage of AnnexinV^+^ PI^+^ (apoptotic) A549 cells in the 4 treatment groups based on FACS analysis. **(D)** Total number of G2/M phase A549 cells in the 4 treatment groups based on FACS analysis. *P<0.05, **P<0.01 denotes statistical differences in comparison with the control group. #P<0.05 denotes statistical differences in comparison with the IR group. All experiments were performed in triplicate.

Next, we performed FACS analysis to determine the effects of Analgecine on apoptosis and cell cycle. We observed that the apoptotic cell percentages in the combination, control, Analgecine and IR groups were 25.45%±1.08, 7.67%±0.08, 11.29%±0.38, and 16.69%±2.45, respectively (Figure 5A, 5C; P<0.05 compared to control). Moreover, FACS analysis of cell cycle showed that Analgecine alone or in combination with IR induced cell cycle arrest in the G2/M phase. The percentage G2/M phase cells in the combination, control, Analgecine and IR groups were 37.4%±0.17, 19.23%±2.08, 24.1%±1.32, and 34.07%±1.55, respectively (Figure 5B, 5D; P<0.05 compared to control).

### Analgecine treatment increases Bax, but reduces Bcl2 levels in A549 cells

**Figure 6:**
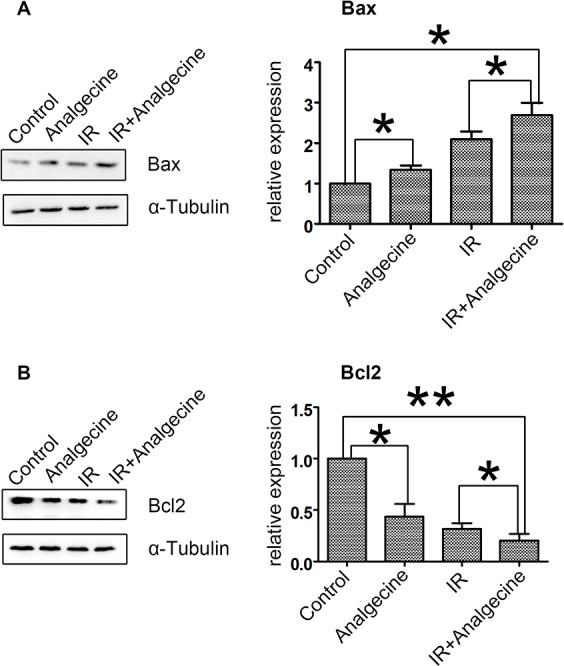
Effect of Analgescine plus IR treatment on Bax and Bcl2 levels in A549 cells **(A-B)** Western blot analysis and quantification of pro-apoptotic Bax, anti-apoptotic Bcl2 proteins is shown. A549 cells treated with Analgecine alone or in combination with IR showed increased Bax and decreased Bcl2 compared to control. *P<0.05, **P<0.01 denote statistical differences indicated. The results are expressed as mean±SD of three independent experiments.

Next, we analyzed the expression of apoptosis related proteins, Bax and Bcl-2 in the 4 groups of A549 cells. We observed that treatment with Analgecine or IR increased pro-apoptotic Bax levels (Figure 6A). Moreover, Bax levels were higher in the combination group compared to the control and Analgecine groups (P<0.05). Conversely, Bcl2 expression was decreased in the combination group compared to the control group (P<0.01; Figure 6B). These data suggested that Analgecine enhanced antitumor response by activating apoptosis pathways.

Further, we observed increased Bax mRNA and reduced BCl-2 mRNA in all treatment groups compared to the control (Supplementary Figure 1).

### Analgecine reduces G2/M phase regulatory proteins

**Figure 7:**
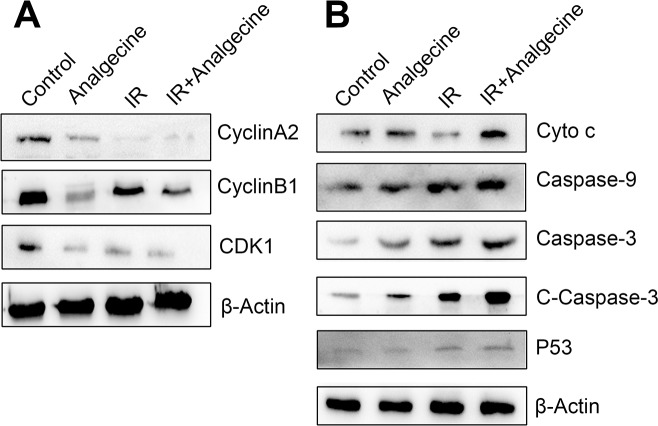
Western blot analysis of G2/M checkpoint and apoptosis pathway proteins in Analgecine and IR treated A549 cells **(A)** Western blot analysis shows decreased cyclinA2, cyclinB1, CDK1 levels in A549 cells treated with Analgecine alone or in combination with IR correlating with G2/M arrest. **(B)** Western blot analysis shows increased cytochrome c, caspase-9, caspase-3, cleaved caspase-3, p53 levels in A549 cells treated with Analgecine plus IR resulting in enhanced activation of apoptotic signaling. Cyto c: cytochrome c; C-caspase-3: cleaved caspase-3; IR: ionizing radiation.

Since FACS analysis demonstrated that Analgecine enhanced G2/M phase arrest, we analyzed the expression of G2/M phase associated proteins, namely, cyclinA2, cyclinB1 and CDK1 by western blotting. Western blot analysis demonstrated that Analgecine treatment, especially in combination with IR decreased cyclinA2, cyclinB1 and CDK1 levels, thereby resulting in G2/M arrest (Figure 7A).

### Analgecine activates apoptotic signaling pathway

Next, we analyzed the status of apoptotic signaling pathways upon Analgecine treatment in the 4 groups of A549 cells. Western blot analysis showed increased cytochrome c, caspase-3, cleaved caspase-3, caspase-9 and p53 levels in the Analgecine and combination treatment groups (Figure 7B). This further demonstrated that Analgecine treatment activated the apoptotic signaling pathways.

## DISCUSSION

Analgecine is widely used in China to alleviate chronic pain with no reported adverse effects [12]. However, there have been no studies regarding its use in combination with radiotherapy. Therefore, we analyzed the effect of a combination of radiotherapy with Analgecine on the mechanisms underlying the antitumor responses using the LLC model *in vivo* and the A549 cell line model *in vitro*.

The *in vivo* LLC mouse model demonstrated that Analgecine enhanced tumor growth inhibition in combination with IR by enhancing apoptosis and inhibiting tumor cell cycle. We observed that the combination treatment increased IL-6, IL-12, and IFN-γ expression and decreased TGF-β1 and IL-10 expression [17–19]. Further, treatment with Analgecine alone or in combination with IR increased pro-apoptotic proteins like cytochrome c, caspase-3, cleaved caspase-3, caspase-9, p53 and Bax, whereas the anti-apoptotic protein Bcl2 was decreased. This indicated that Analgecine improves antitumor response with IR by enhancing apoptotic signaling [20–23].

*In vitro* experiments with A549 NSCLC cells further demonstrated that Analgecine in combination with IR decreased cell viability and enhanced radiosensitivity. Further, Analgecine alone or in combination with IR induced cell apoptosis and G2/M phase cell cycle arrest. This was further corroborated by significantly decreased cyclinB1 and CDK1 in the combination treatment, thereby inducing G2/M phase arrest. Previous studies have shown that targeting apoptotic pathways is a crucial strategy in antitumor therapy [24]; Also, the G2/M cell cycle phase is most sensitive to IR [25]. Based on these results, we postulated that Analgecine in combination with radiotherapy enhanced the antitumor response by activating apoptotic pathways and inducing G2/M phase arrest.

This study has some limitations. This study was performed with a single cell line *in vitro* and needs to be analyzed in other NSCLC cell lines. Moreover, comprehensive analysis of various signaling pathways is necessary to understand the modulation of other signaling pathways by Analgecine to enhance the antitumor response to IR.

In conclusion, this study demonstrates that Analgecine promotes the IR-induced antitumor response by inducing apoptosis, G2/M cell cycle arrest. We postulate that Analgescine has potential in clinical applications for lung cancer.

## MATERIALS AND METHODS

### Cell lines and culture

Human lung cancer cells (A549 and LLC) were purchased from the Cell Resource Center of the Chinese Academy of Sciences (Shanghai, People's Republic of China). The A549 cells were cultured in complete RPMI-1640 medium, whereas the LLC cells were grown in Dulbecco's modified Eagle's medium (Corning, NY, USA) supplemented with 10% fetal bovine serum (Gibco, Grand Island, NY, USA) in a sterile incubator maintained at 37°C with 5% CO_2_.

### Analgecine

Analgecine (1.2U/ml) was purchased from Vanworld Pharmaceutical Rugao (Jiangshu, China) and dissolved in sterilized phosphate-buffered saline (PBS; Corning, NY, USA) to generate a stock solution at a concentration of 0.12U/ml. The Analgecine dose was chosen based on the reported clinical dose [12].

### Xenograft mouse model

Six- to eight-week-old female C57BL/6 mice were purchased from the Shanghai Experimental Animal Center and raised in pathogen-free conditions. All animal experiments were approved by the Institutional of Animal Care and Use Committee of Jinshan Hospital, Fudan University. The mice were subcutaneously injected with 5×10^6^ LLC cells into the right flank. After tumors grew for two weeks, 32 tumor-bearing mice were randomly assigned to one of the 4 groups: (1) control group; (2) Analgecine group; (3) IR group; and (4) IR plus Analgecine (combination) group. Treatments were initiated two weeks after LLC cell implantation (day 1 treatment). The control group was injected with an equal volume of normal saline. The Analgecine and the combination group mice were administered Analgecine three days prior to IR. Each tumor was irradiated with 3Gy X-rays using a Trilogy linear accelerator (Varian Medical Systems, CA, USA) on five consecutive days starting on day 4, and Analgecine was injected 2h before IR in the combination group during the five days of IR. We continued to inject Analgecine after all IR sessions for three days. To assess tumor growth, tumor diameters were measured with calipers thrice every week. The tumor volume was calculated using the formula V= (A×B^2^)×0.5, where A is the longest dimension, B is the perpendicular dimension [25]. Similar treatment protocol was followed for survival analysis (8 mice per group).

### Terminal deoxynucleotidyl transferase dUTP nick end labeling (TUNEL) assay

TUNEL assay was performed on harvested xenograft tumors to analyze apoptosis. Tumors were fixed in 4% paraformaldehyde and embedded in paraffin. Then, TUNEL assay was performed on the tumor specimens according to instructions with the TUNEL assay kit (KeyGEN, Nanjing, China).Then, the specimens were incubated with 4′,6-diamidino-2-phenylindole (DAPI; blue fluorescence) to stain nuclei. The green fluorescence represented TUNEL-positive cells, which were randomly counted in 10 low-power fields (100×) for each sample.

### Plasma cytokine analysis

To determine the effects of Analgecine on the immune response, we detected transforming growth factor (TGF)-β expression using the TGF-β Magnetic Bead Kit (Millipore, Billerica, MA, USA) and IL-6, IL-10, IL-12, tumor necrosis factor (TNF)-α and interferon (IFN)-γ expression using the Mouse Cytokine/Chemokine Magnetic Bead Panel (Millipore, Billerica, MA, USA) with mouse plasma according to the manufacturer's instructions. The protocol for preparation of plasma samples were showed in Supplementary Materials.

### *In vitro* experimental design

To determine the effects of Analgecine in combination with IR *in vitro*, the A549 cell line was divided into the following four groups: (1) control group treated with equal amounts of solvents, normal saline; (2) Analgecine group treated with Analgecine (0.12 U/ml); (3) IR group treated with 10Gy X-ray IR; and (4) IR plus Analgecine (combination) group, treated first with Analgecine (0.12U/ml) followed by a cycle of radiotherapy.

### Cell counting kit-8 (CCK-8) cell viability assays

A549 cells (3×10^3^) were seeded onto 96-well culture plates and grown overnight. After all treatments, the CCK-8 assay (Dojindo, Kumamoto, Japan) was performed according to the manufacturer's protocol. The plates were incubated for 1-4h and then the absorbance was measured at 450 nm in a plate reader ((BioTek Epoch, Winooski, VT, USA).

### Colony-formation assay

A549 cells were seeded in 6-well plates. After 24 hours, the cells in Analgecine and combination groups were treated with 0.12U/ml Analgecine. Then, the IR and combination groups were irradiated with X-ray doses ranging from 0 to 10Gy, and fresh medium was added immediately. The cells were then trypsinized, counted, and seeded (800 cells per well) for colony formation in 60-mm dishes. The cells were incubated for 10 to 14 days for colony formation. The colonies were fixed with 4% paraformaldehyde and stained with crystal violet. Colonies of ≥50 cells were considered clonogenic survivors. The data were analyzed with single-hit, multi-target models [26]. The radiobiological parameters were calculated such as D0 (the mean radiation dose induced 63% cells to death), N(the extrapolation number) and sensitivity enhancement ratio(SER, SER=the control D0/ the treatment D0).

### Apoptosis and cell cycle assays by flow cytometry

A549 cells were seeded into 6-well plates and treated with Analgecine alone or in combination with IR for 24h. Then, the cells were stained with the Annexin V/ propidium iodide (PI) kit (BD Biosciences, San Jose, CA, USA). Another set of cells were stained with a propidium iodide staining kit (BD Biosciences, San Jose, CA, USA) for cell cycle analysis. Both staining protocols were performed according to the manufacturer's instructions. The flow cytometry assay was analyzed using the flow cytometer (Beckman, Miami, FL, USA). The experiments were performed in triplicate and repeated thrice.

### Western blot analysis

Whole protein extracts were prepared by SDS lysis buffer (20 mM Tris-HCl (pH 7.5) 150mM NaCl, 1mM Na2EDTA. 1mM EGTA, 1% Triton, 2.5mM sodium pyrophosphate, 1mM β-glycerophosphate, 1mM Na_3_VO_4_, 1μg/ml leupeptin) containing 1% phenylmethanesulfonyl fluoride (KeyGEN, Nanjing, China). The protein concentrations were detected using a BCA Protein Assay Reagent Kit (Pierce, Rockford, IL, USA). Equal protein from all samples were separated by 10% or 12% SDS-PAGE (120V for 90 minutes) and transferred to polyvinylidene fluoride membranes (Millipore, Billerica, MA, USA). Membranes were probed with the primary antibodies (see Supplementary Table 1) followed by appropriate secondary antibodies, horseradish peroxidase-conjugated goat anti-rabbit IgG or anti-mouse IgG (Millipore; Billerica, MA, USA, both at 1:5000). The protein bands were detected via enhanced chemiluminescence (Millipore, Billerica, MA, USA) and quantified using Gel Imaging System with GIS ID Analysis Software v4.1.5 (Tanon, Shanghai, China).

### Quantitative RT-PCR (qRT-PCR)

Total RNA was extracted from all 4 treatment groups of A549 cells with the Axyprep Multisource Total RNA miniprep Kit (Axygen, Union City, CA, USA) and quantified. Then, 1μg RNA from all RNA samples was reverse transcribed into cDNA with the PrimeScript RT Master Mix (TaKaRa, Dalian, China). QPCR was performed with a SYBR Premix Ex Taq (Tli RNaseH Plus; TaKaRa) according to the manufacturer's protocols in a Applied Biosystem 7300 (Applied Biosystems, Foster city, CA, USA). The target and control gene primers are shown in Supplementary Table 2. The target gene levels were normalized to β-Actin levels according to the ΔΔCT method.

### Statistical analyses

The SPSS software version 23.0 (SPSS, Chicago, IL, USA) was used for the statistical analysis. The quantitative data were expressed as mean±SD. Survival curves were constructed with the Kaplan-Meier method and analyzed by log-rank test. When comparing two treatment groups, P values were assessed using 2-tailed unpaired Student's t-tests. A P-value of less than 0.05 was considered significant.

## SUPPLEMENTARY MATERIALS FIGURE AND TABLES



## Abbreviations

FITC: fluorescein isothiocyanate
IFN: interferon
IL: interleukin
IR: ionizing radiation
NSCLC: non-small-cell lung cancer
PBS: phosphate-buffered saline
SER: sensitivity enhancement ratio
TGF: transforming growth factor
TNF: tumor necrosis factor
