# Supplementary material for: Analgecine enhances the anti-tumor response of radiotherapy by increasing apoptosis and cell cycle arrest in non-small cell lung cancer
Source: Oncotarget. 2017 Aug 7;8(46):80730–40. doi: 10.18632/oncotarget.19968 (PMC5655235; doi:10.18632/oncotarget.19968)
Supplement: Supplementary file 1 [file oncotarget-08-80730-s001.pdf]

Analgecine enhances the anti-tumor response of radiotherapy by increasing apoptosis and cell cycle arrest in non-small cell lung cancer

SUPPLEMENTARY MATERIALS

Preparation of plasma samples

1. Plasma collection using EDTA as an anti-coagulant is recommended. Centrifuge for 10 minutes at 1000×g within 30 minutes of blood collection. Remove plasma and assay immediately or aliquot and store samples at ≤ -20°C.
2. Avoid multiple(>2) freeze/thaw cycles
3. When using frozen samples, it is recommended to thaw the samples completely, mix well by vortexing and centrifuge prior to use in the assay to remove particulates.
4. Plasma samples should be diluted 1:2 in the Assay Buffer provided in the kit(i.e. One part plasma sample into one part Assay Buffer). For example, in a tube, 30 µl of plasma may be combined with 30 µl of Assay Buffer. When further dilution beyond 1:2 is required, use serum Matrix as the diluent.

(Mouse Cytokine/Chemokine Magnetic Bead Panel , Cat. # MCYTOMAG-70K; TGF-β Magnetic Bead Kit, Cat. # TGFBMAG-64K-03 ; Millipore, Billerica, MA, USA)

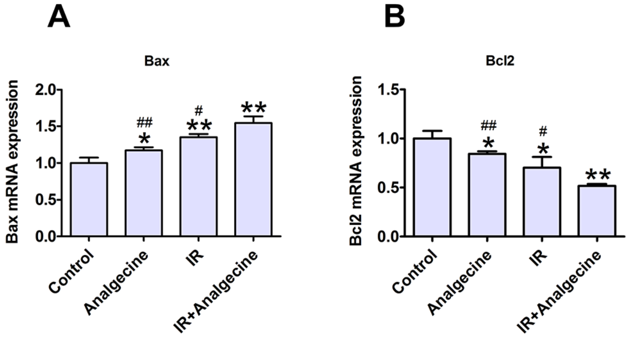

Supplementary Figure 1: Effect of Analgecine on Bax and Bcl2 mRNA levels. (A) Quantitative RT-PCR analysis showed that Bax gene expression was increased in Analgecine (P<0.05), IR (P<0.01), combination (P<0.01) treatment groups compared to the control. **(B)** Quantitative RT-PCR analysis showed that Bcl2 gene expression was decreased in Analgecine (P<0.05), IR (P<0.05) and combination (P<0.01) groups compared to the control. Note: The primer sequences of Bax, Bcl2 were showed in supplementary Table 2. *P<0.05, **P<0.01 denote statistical differences compared to the control; #P<0.05, ##P<0.01 denote statistical differences compared to combination group. IR: ionizing radiation.

Supplementary Table 1: The details of the primary and second antibodies for western blot assays

Antibody	Catalog number	Source	Dilution	Company
Bax	2772	Rabbit	1:1000	Cell Signaling Technology, Danvers, MA, USA
Bcl2	2870	Rabbit	1:1000	Cell Signaling Technology, Danvers, MA, USA
CyclinA2	4656	Rabbit	1:1000	Cell Signaling Technology, Danvers, MA, USA
CyclinB1	55004-1-AP	Rabbit	1:3000	Proteintech, Wuhan, China
CDK1	77055	Rabbit	1:1000	Cell Signaling Technology, Danvers, MA, USA
Cytochrome c	4280	Rabbit	1:1000	Cell Signaling Technology, Danvers, MA, USA
Caspase-9	9502	Rabbit	1:1000	Cell Signaling Technology, Danvers, MA, USA
Caspase-3	9665	Rabbit	1:1000	Cell Signaling Technology, Danvers, MA, USA
Cleaved caspase-3	9661	Rabbit	1:1000	Cell Signaling Technology, Danvers, MA, USA
p53	2524	Mouse	1:1000	Cell Signaling Technology, Danvers, MA, USA
α-Tubulin	HRP-66031	-	1:5000	Proteintech, Wuhan, China
β-Actin	HRP-60008	-	1:5000	Proteintech, Wuhan, China

Supplementary Table 2: The primer sequences of Bax, Bcl2

Target gene	Primers
Bax	
Forward Primer	5’-GAGCAGATCATGAAGACAGG-3’
Reverse Primer	5’-CCTCTGCAGCTCCATGTTAC-3’
Bcl2	
Forward Primer	5’-AACATCGCCCTGTGGATGAC-3’
Reverse Primer	5’-AGAGTCTTCAGAGACAGCCAGGAG-3’
β-Actin	
Forward Primer	5’- CATTGCCGACAGGATGCAG-3’
Reverse Primer	5’- CTCGTCATACTCCTGCTTGCTG-3’
